# Supplementary material for: Sea lamprey enlightens the origin of the coupling of retinoic acid signaling to vertebrate hindbrain segmentation
Source: Nat Commun. 2024 Feb 20;15:1538. doi: 10.1038/s41467-024-45911-x (PMC10879103; doi:10.1038/s41467-024-45911-x)
Supplement: Supplementary file 1 — Supplementary Information [file 41467_2024_45911_MOESM1_ESM.pdf]

Supplementary information for article:

Sea lamprey enlightens the origin of the coupling of retinoic acid signaling to vertebrate hindbrain segmentation

Alice M. H. Bedois[1], Hugo J. Parker[1], Andrew J. Price[1], Jason A. Morrison[1], Marianne E. Bronner[2] and Robb Krumlauf[1,3,*]

[1] Stowers Institute for Medical Research, Kansas City, Missouri 64110, USA

[2] Division of Biology and Biological Engineering, California Institute of Technology, Pasadena, CA, 91125, USA

[3] Department of Anatomy and Cell Biology, Kansas University Medical Center, Kansas City, Kansas 66160, USA

*Corresponding author and contact details:
Robb Krumlauf, Stowers Institute for Medical Research, 1000 East50th, Kansas City, MO 64110, USA, Email: rek@stowers.org; Tel:1-816-926-4051

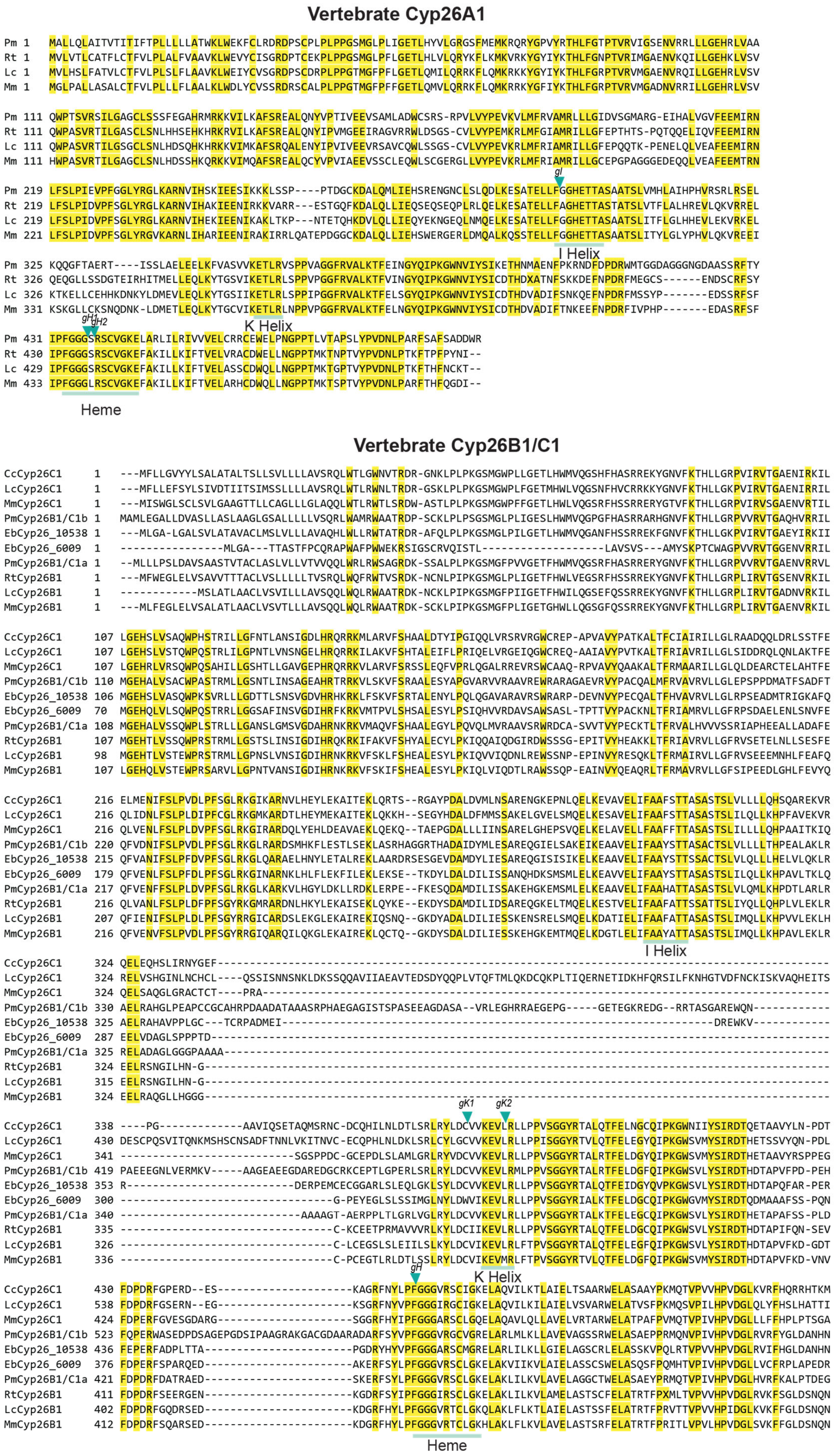
Vertebrate Cyp26A1
Pm
Rt
Lc
Mm
gI
I Helix
K Helix
gH1
gH2
Heme
Vertebrate Cyp26B1/C1
CcCyp26C1
LcCyp26C1
MmCyp26C1
PmCyp26B1/C1b
EbCyp26_10538
EbCyp26_6009
PmCyp26B1/C1a
RtCyp26B1
LcCyp26B1
MmCyp26B1
I Helix
gK1
gK2
K Helix
gH
Heme

Supplementary Figure 1: Vertebrate Cyp26 protein alignments. Protein alignments of vertebrate Cyp26A1 and vertebrate Cyp26B1/C1. The yellow highlighting represents the identity between sequences with a 95% threshold for sequence identity. Conserved K, I helices and Heme domains are indicated. Cutting sites of the gRNAs used for the CRISPR/Cas9 experiments are indicated with a teal arrow. Cc, Carcharodon carcharias; Eb, *Eptatretus burgeri*; Lc, *Latimeria chalumnae*; Mm, *Mus musculus*; Pm, *Petromyzon marinus*; Rt, *Rhincodon typus*.

Vertebrate Aldh1a1 and Aldh1a2

```
PmAldh1a1/a2b 1   MA-----QRGD-----GVGRVGEGAGGPIPTPVADPQMKYTQLFINNEWQEAASGRKFDVTNPSTGQLLCSVAEADEEDVDRAVRAARAAFRLGSPWRRMDPSARGRLLA
EbAldh1a1     1   MSEVASRTDGVL------SNAFDGLSISPPSPVMDLQIKYTKIFINNSWHDSLNGDTFPTVDPSNGCKLCDVQEGREVDVELAVKAAREAFRPGSPWRRLDASERGRLLN
PmAldh1a1/a2a 1   -MSGGAEVPGGDAAQAGLAGLLASLPGVPPSPVRDLRARYTKIFIGNEWRDSISGRTFPTFDPASGEKLCDVQEGDHEDVNVAVVAAREAFVLGSPWRRMDASDRGVLLS
RtAldh1a2     1   MTSSEIEIPSEVKT--DPAA-LIASLQLLPSPTPNLEIQHTKLFINNEWHHSVSGKTFSTYNPSTGEKICDVQEADKADVDKAVQAARIAFSPGSVWRRMDASERGRLID
LoAldh1a2     1   MTSSKIELPGEVKT--DPAA-LVASLHLVPSPVPNLEIKHTKIFINNEWQNSVSGKTFPTYNPSNGEKICDVQEAEKADVDKAVQAARLAFSLGSVWRIMDASERGRLLD
LcAldh1a2     1   MTSSKIELPGEVKT--DPAA-LMASLHLLPSPIPNLEIKFTKIFINNEWQNSESGKTFPVYNPSTGEQICEVQEADKLDTDKAVQAARLTFSLGSVWRRMDASERGRLLN
MmAldh1a2     1   MTSSEIAMPGEVKA--DPAA-LMASLQLLPSPTPNLEIKYTKIFINNEWQNSESGRVFPVCNPATGEQVCEVQEADKVDIDKAVQAARLAFSLGSVWRRMDASERGRLLD
MmAldh1a1     1   ------------------M-SSPAQPAVPAPLADLKIQHTKIFINNEWHNSVSGKKFPVLNPATEEVICHVEEGDKADVDKAVKAARQAFQIGSPWRTMDASERGRLLN
RtAldh1a1     1   MS----A----PAA--DDES-PAPAPRYVPAPLTELQIKYTKIFINNEWHNSSSGKKFTTYNPATGEKICDIEEGDKVEVDKAVKAAREAFQIGSTWRRMDASGRGRLLH
LcAldh1a1     1   MS----ATPCESQN--SDLK-PAVVPPGLPVPVSNLEIKYTKIFINNDWHDSSNGKRFPTYNPATGEKICEVEEGDKEDVDKGVKAAREAFQLGSLWRTMDASERGKLLN

PmAldh1a1/a2b 101 RLADLVERDRALLSTLECLDAGKPFLTTYFVDLDGVIRTLRYYAGWADKVQGRTVPVDGDFISYTRHEPIGVCGQIIPWNFPLLMLAWKLAPALCCGNTVVVKPAEQTPL
EbAldh1a1     105 RLAELVERDVTLLSTLESLDSGKPFLHTFFVDMMGIVKTLRYYAGWCDKVQGRTIPVDGEYFTYTRHEPVGVCGQIIPWNFPLMMFVWKIAPALCCGCTIVIKPAEQTPL
PmAldh1a1/a2a 110 RLADLVERERTTLATLESMDSGKPFLPAFFVDVTGAVKTLRYFAGWCDKIQGRTIPVDGEYFTFTRHEPIGVCGQIIPWNFPLLMFIWKIAPALSCGNTVVVKPAEQTPL
RtAldh1a2     108 KLADLVERDRAHLATLESLDSGKPFLQAYYVDLQGVIKTLRYFAGWTDKIHGKTIPVDGDYFTFTRHEPIGVCGQIIPWNFPLLMFAWKIVPALCCGNTVVIKPAEQTPL
LoAldh1a2     108 KLANLVERDRVLLATLESLNSGKPFLQAYYVDLQGTIKTLRYFAGWADKIHGMTIPMDGDYFTFTRNEPIGVCGQIVPWNFPLLMVAWKIAPALCCGNTVVLKPAEQTPL
LcAldh1a2     108 KLADLVERDSTLLATMESLNGGKPFLQAFYVDLQGVIKTLRYFAGWADKIHGVTIPVDGDYFTFTRHEPIGVCGQIIPWNFPLLMFAWKIAPALCCGNTVVIKPAEQTPL
MmAldh1a2     108 KLADLVERDRATLATMESLNGGKPFLQAFYIDLQGVIKTLRYYAGWADKIHGMTIPVDGDYFTFTRHEPIGVCGQIIPWNFPLLMFTWKIAPALCCGNTVVIKPAEQTPL
MmAldh1a1     91  KLADLMERDRLLLATMEALNGGKVFANAYLSDLGGCIKALKYCAGWADKIHGQTIPSDGDIFTYTRREPIGVCGQIIPWNFPMLMFIWKIGPALSCGNTVVVKPAEQTPL
RtAldh1a1     100 KLADLMERDRVILSTIESIDSGKLFLHAYFVDLDGSIKTLRYYAGWADKIQGRTIPVDGDYLTFTRHEPIGVCGQIIPWNFPLLMFTWKLAPALCCGNTVVIKPAEQTPL
LcAldh1a1     104 KLADLVERDQQMLSTIESVDSGKPFLSAYTIDLTATIKTLRYYAGWTDKIQGRTIPVDGDYFTFTRHEPIGVCGQIIPWNFPLLMFSWKIAPALCCGNTVVIKPAEETPL

                                                                                 g1      Glu                   g2 Cys
PmAldh1a1/a2b 211 SALHVCSLILEAGFPPGVVNMLPGFGPTAGAAIARHVDVDKVAFTGSTEVGKLIQQAAGASNLKRVTLELGGKSPNIVFADADLDLAVEQAHQGSFFNQGQCCTAGSRVF
EbAldh1a1     215 TALHMGALIKEAGFPAGVVNIVPGYGPTAGAAIARHPDINKVSFTGSTEVGKLIQEAAGKSNLKRVTLELGGKSPIIVFPDADLDVAVKETHEAVFFNQGQCCTAGSRLF
PmAldh1a1/a2a 220 TALHVGALIAEAGFPPGVVNIVPGFGPTAGAAIVQHPDIDKIAFTGSTEVGKLIQEEAGKSNLKRVTLELGGKSPIIVFADADLDAAVEQAHQGVFWNQGQCCTAGSRVY
RtAldh1a2     218 TALYMGALIIEAGFPPGVVNIVPGFGVTAGAAIASHMDIDKIAFTGSNEVGKLIQEAAGKSNLKRVTLELGGKSPNIIFADADLDYAVEQAHQGVFFNQGQCCTAGSRIY
LoAldh1a2     218 SCLYMGALIKEAGFPPGVVNILPGFGPTAGAAIANHMGIDKIAFTGSTEVGKLIQEAAGRSNLKRVTLELGGKSPNIIFADADLDYAVEQAHQGVFFNQGQCCTAGSRVF
LcAldh1a2     218 SALYMGALIKEAGFPPGVVNILPGYGPTAGTAIAAHMGIDKVAFTGSTEVGKLIQEAAGRSNLKRVTLELGGKSPNIIFADADLEHAVEQAHQGVFFNQGQCCTAGSRIY
MmAldh1a2     218 SALYMGALIKEAGFPPGVVNILPGYGPTAGAAIASHIGIDKIAFTGSTEVGKLIQEAAGRSNLKRVTLELGGKSPNIIFADADLDYAVEQAHQGVFFNQGQCCTAGSRIF
MmAldh1a1     201 TALHLASLIKEAGFPPGVVNIVPGYGPTAGAAISSHMDVDKVAFTGSTQVGKLIKEAAGKSNLKRVTLELGGKSPCIVFADADLDIAVEFAHHGVFYHQGQCCVAASRIF
RtAldh1a1     210 TALYMGALVKEAGFPPGVVNIVPGYGCTAGATITSHMDIDKVAFTGSTEVGKLIQEAAGKSNLKRVTLELGGKNPNIIFADADLDFAVEQAHHALFFHQGQCCLAGSRIF
LcAldh1a1     214 TALYLGALIKEAGFPPGVVNIIPGFGPTAGAAISQHMDIDKVAFTGSTEVGKLIKQAAGKSNLKRVTLELGGKNPNIILADTDLDYAVEQAHYGLFFNQGQCCLAGSRIF

PmAldh1a1/a2b 321 VQAPVYDEFVRRTVERARRRALGDPLRPGTDQGPQVDQTQFDKVLELVESGKTEGAHVACGGGADTVAGALFIQPTVFTDVQDHMRIVTEEIFGPVQMILKFDTVEEVLE
EbAldh1a1     325 VHESIYRDFVRRSVECARQRRLGNPLHPSTQQGPQIDETQRKRILELIESGVREGARLECGGCTWGD-QGYFLEPTVFSDVTDDMRIACEEIFGPVQQIMSFSTSEEVLA
PmAldh1a1/a2a 330 VEDPVYDEFVRRSAQRARVRRVGHPFCPSTEHGPQIDEKQRSKILELVQSALEEGAHLECGGVACEG-RGFYVQPTVFSDVPDSARITREEIFGPVQLIMRFDSLEDVVA
RtAldh1a2     328 VEEPIYDEFVRKSTERAQRRTVGNPFDPATEQGPQIDKLQCDKVLELVLSGIAEGAKLECGGKSLGK-KGFFIEPTVFSNVTDDMRIAKEEIFGPVQPIIKFKTMEEVVE
LoAldh1a2     328 VEEPVYEEFVRKSVERAKRRVVGSPFDPTTEQGPQISKEQQSQILELIQSGISEGAKLECGGKAIGR-KGFYIEPTVFSNVRDEMRIAKEEIFGPVQQIMKFKSIDEVIE
LcAldh1a2     328 VEEPIYEEFVRRSIERAKRRIVGSPFDPTTEQGPQINKEQYNKILGLIQSGISEGAKLECGGKALGQ-KGFFIEPTVFSNVTDDMRIAKEEIFGPVQQIMKFKSTEEVIE
MmAldh1a2     328 VEESIYEEFVKRSVERAKRRIVGSPFDPTTEQGPQIDKKQYNKVLELIQSGVAEGAKLECGGKGLGR-KGFFIEPTVFSNVTDDMRIAKEEIFGPVQEILRFKTMDEVIE
MmAldh1a1     311 VEESVYDEFVKRSVERAKKYVLGNPLTPGINQGPQIDKEQHDKILDLIESGKKEGAKLECGGGRWGN-KGFFVQPTVFSNVTDEMRIAKEEIFGPVQQIMKFKSVDDVIK
RtAldh1a1     320 VEEPVYKEFVCKSISLAQKHVIGNPLHEAVTHGPQIDKEQYDKILNLIESGKKEGAKLECGGLPWGD-KGFFIQPTVFSEVTDEMRIAKEEIFGPVQQIIKFKTVDEVIK
LcAldh1a1     324 VEESIYEEFVHKSVEQAKKRILGNPLIPGVDQGPQINKQQHDKILELIESGKKEGAKLECGGTSWGD-KGFFIQPTVFSDVTDEMHIAKEEIFGPVQQIMKFKTIDEVIK

PmAldh1a1/a2b 431 RANNTRYGLAAAVFTRDLDTALTLAAGLQAGTVWVNCYNVIQAQTAFGGFKMSGNGREMGEYGLQEYTEVKTVTIRVPKKSS
EbAldh1a1     434 RANDSYYGLAAGVFSKDLDTVLHVAAALQAGTVWVNCYNAVNCQSPFGGFKMSGNGREMGEYGLQEYTEVKTVTIRLPNKML
PmAldh1a1/a2a 439 RANASPYGLVGAVFTRDLERALAVSAAMQAGTVWINCYNAMNAQSPFGGYKMSGNGREMGEYGLQEYTEVKTVTIKISQKNS
RtAldh1a2     437 RANKSQFGLVAAVFTKDIDKALTVSAAMQAGTVWINCYNALNVQSPFGGFKMSGIGREMGEYGLREYTEIKTVTVRIQQKNS
LoAldh1a2     437 RANNSEYGLTAGVFTRDINKAMTVSTAMQAGTVWINCFNALSAQCPFGGFKMSGIGREMGEYGLREYSEVKTITIKVPQKNS
LcAldh1a2     437 RANNSDFGLVAAVFTNDLNKALTVSSAMQAGTVWGEC-------------------------GLREYSEVKTVTIKIPQKNS
MmAldh1a2     437 RANNSDFGLVAAVFTNDINKALMVSSAMQAGTVWINCYNALNAQSPFGGFKMSGNGREMGEFGLREYSEVKTVTVKIPQKNS
MmAldh1a1     420 RANNTTYGLAAGLFTKDLDKAITVSSALQAGVVWVNCYMMLSAQCPFGGFKMSGNGRELGEHGLYEYTELKTVAMKISQKNS
RtAldh1a1     429 RANNTHYGLVAAVFTKDINKAFTVASALQAGTVWVNCYNAMHVQSPFGGFKMSGNGREMGEYGLQEYTEIKTITIKIPQKNS
LcAldh1a1     433 RANNTHYGLVAGVFTKDLNKAMTIASSLQTGTVWVNCYNAMTPQCPFGGFKMSGNGREMGEYGLQEYTEIKTITIKLSQKNS
```

Supplementary Figure 2: Vertebrate Aldh1a protein alignments. Protein alignments of sea lamprey (Pm) Aldh1a1/a2a and Aldh1a1/a2b with Aldh1a1 and Aldh1a2 from various jawed vertebrates. The yellow highlighting represents the identity between sequences with a 95% identity threshold. Conserved Glutamic acid (Glu) and Cysteine (Cys) domains are indicated. Cutting sites of the gRNAs used for the CRISPR/Cas9 experiments are indicated with a blue arrow. Eb, *Eptatretus burgeri;* Lc, *Latimeria chalumnae*; Lo *Lepisosteus oculatus;* Mm, *Mus musculus;* Pm, *Petromyzon marinus*; Rt, *Rhincodon typus.*

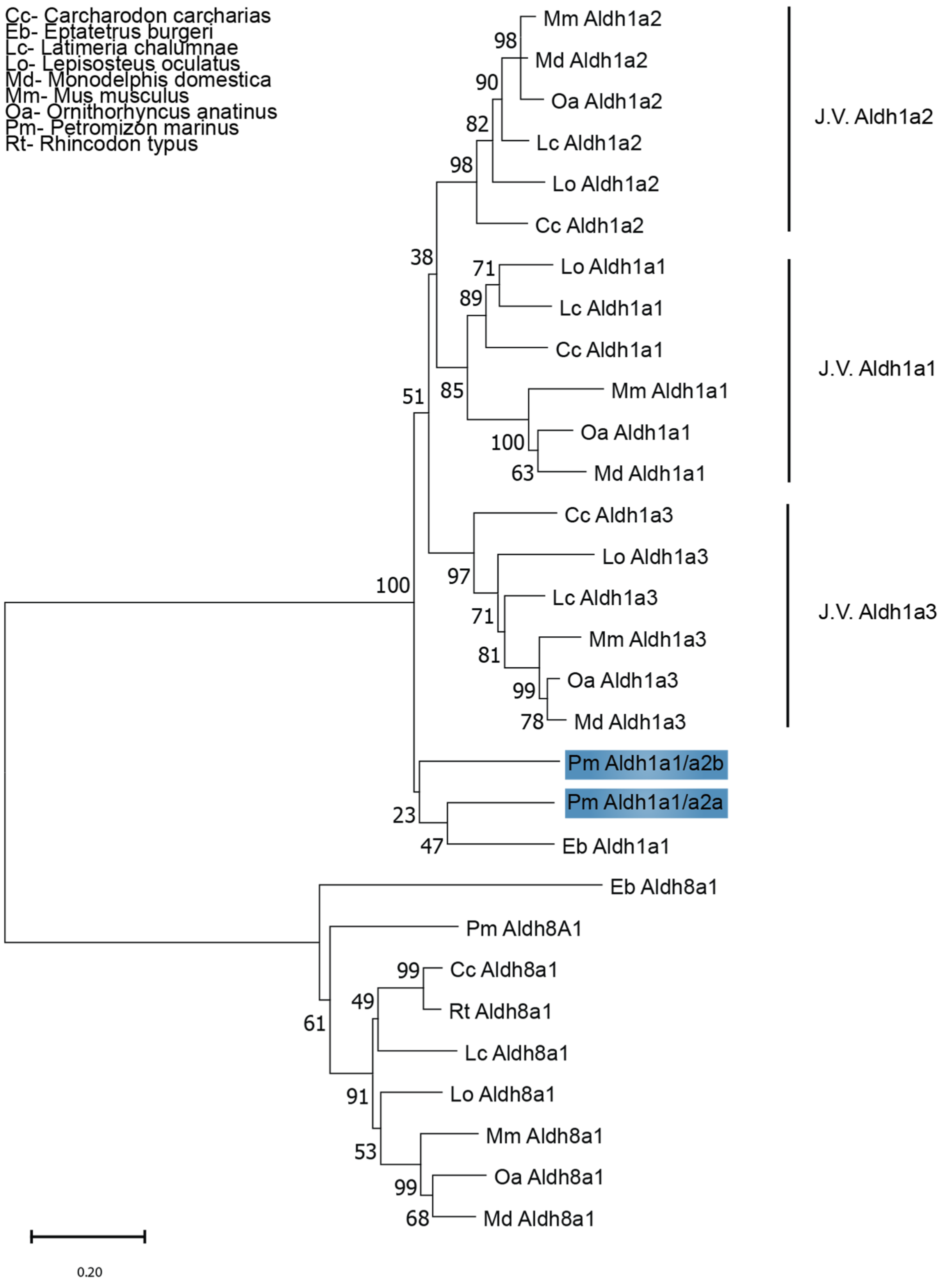

Supplementary Figure 3: Vertebrate *Aldh1a* Phylogeny. Phylogenetic analysis of the vertebrate *Aldh1a* complement with *Aldh1a8* used as an outgroup. Jawed Vertebrate (J.V.) *Aldh1a1/Aldh1a2/Aldh1a3* clades are indicated with black lines. Trees were generated by Maximum Likelihood using the WAG model with 500 iterations for bootstrap testing, and the resulting supporting value for each node is shown as a percentage. A scale bar for the evolutionary distance is indicated below each tree. Species name abbreviations are indicated on the top left of the panel.

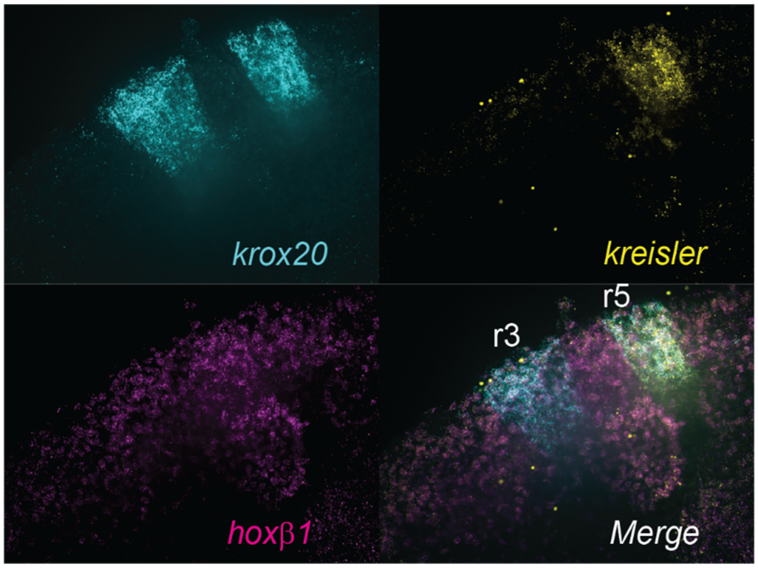

Supplementary Figure 4: HCRv3 development and validation in the sea lamprey. HCR panel representing a high-power (40X) image of *krox20* (cyan), *kreisler* (yellow) and *hoxβ1* (magenta) with their merged expression. This image was obtained after optimizing and adapting our HCRv3 protocol to be able to visualize hindbrain segments. We then used this protocol with this combination of genes to understand the changes in hindbrain segmentation in Talarazole vs. DMSO embryos (**Fig. 6a**).

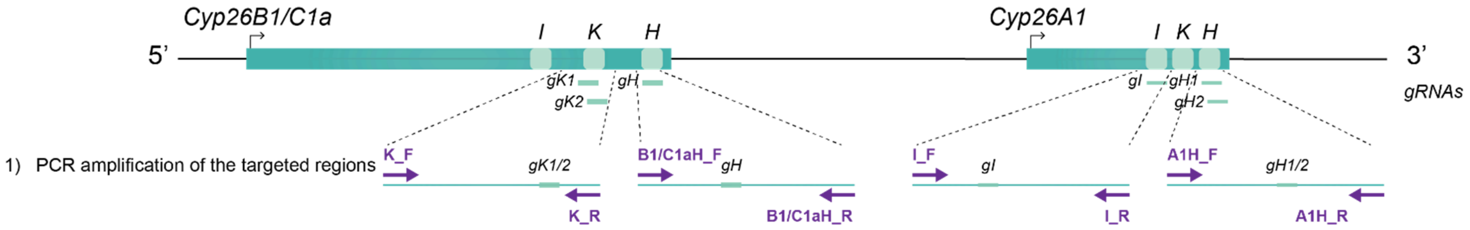

2) Sequencing of the targeted regions

```
Ctrl_C1H    GGAGCGTCCTCTACAGCATCCGCGACACGCACGAGACGGCGCCGGCCTTCAGCTCGCCGC  60
Cr_C1H_1    GGAGCGTCCTCTACAGCATCCGCGACACGCACGAGACGGCGCCGGCCTTCAGCTCGCCGC  60
Cr_C1H_2    GGAGCGTCCTGTACAGCATCCGCGACACGCACGAGACGGCGCCGGCCTTCAGCTCGCCGC  60
Cr_C1H_3    GGAGCGTCCTATACAGCATCCGCGACACGCACGAGACGGCGCCGGCCTTCAGCTCGCCGC  60
Cr_C1H_4    GGAGCGTCCTCTACAGCATCCGCGACACGCACGAGACGGCGCCGGCCTTCAGCTCGCCGC  60
Cr_C1H_5    GGAGCGTCCTCTACAGCATCCGCGACACGCACGAGACGGCGCCGGCCTTCAGCTCGCCGC  60
            ********** *************************************************

Ctrl_C1H    TTGACTTCGACCCCGACCGCTTCGACGCGACGCGCGCCGAGGACTCGAAGGAGCGCTTCA  120
Cr_C1H_1    TTGACTTCGACCCCGACCGCTTAGACGCGACGCGCGCCGAGGACTCGAAGGAGCGCTTCA  120
Cr_C1H_2    TTGACTTCGACCCCGACCGCTTCGACGCGACGCGCGCCGAGGACTCGAAGGAGCGCTTCA  120
Cr_C1H_3    TTGACTTCGACCCCGACCGCTTCGACGCGACGCGCGCCGAGGACTCGAAGGAGCGCTTCA  120
Cr_C1H_4    TTGACTTCGACCCCGACCGCTTCGACGCGACGCGCGCCGAGGACTCGAAGGAGCGCTTCA  120
Cr_C1H_5    TTGACTTCGACCCCGACCGCTTCG------------------------------------  69
            ************************

Ctrl_C1H    GCTACCTGCCGTTCGGCGGCGGCGTGCGCAGCTGCCTGGGCAAGGAGCTCGCCAAGCTCA  180
Cr_C1H_1    GCTACCTGCC----GGCGGCGGCGTGCGCAGCTGCCTGGGCAAGGAGCTCGCCAAGCTCA  176
Cr_C1H_2    GCTACCTGC----CGGCGGCGGCGTGCGCAGCTGCCTGGGCAAGGAGCTCGCCAAGCTCA  176
Cr_C1H_3    GCTACCTGC----CGGCGGCGGCGTGCGCAGCTGCCTGGGCAAGGAGCTCGCCAAGCTCA  176
Cr_C1H_4    GCTACCTGCC----GGCGGCGGCGTGCGCAGCTGCCTGGGCAAGGAGCTCGCCAAGCTCA  176
Cr_C1H_5    ---------------GCGGCGGCGTGCGCAGCTGCCTGGGCAAGGAGCTCGCCAAGCTCA  129
                           *********************************************
```

```
Ctrl_A1H    CCCGACCGCTGGATGACCGGCGGCGACGCCGGCGGCGGCAACGGAGACGCGGCATCGTCG  120
Cr_A1H_1    CCCGACCGCTGGATGACCGGCGGCGACGCCGGCGGCGGCAACGGAGACGCGGCATCGTC-  119
Cr_A1H_2    CCCGACCGCTGGATGACCGGCGGCGACGCCGGCGGCGGCAACGGAGACGCGGCATCGTCG  120
Cr_A1H_3    CCCGCCCGCGGGATGACCGGCGGCGCGGCCTGCGGCGGCATCGGAGACGTGGCATTGTCG  120
Cr_A1H_4    CCCGACCGCTGGATGACCGGCGGCGACGCCGGCGGCGGCAACGGAGACGCGGCATCGTC-  119
            **** **** ***************   *** ********* ******** ***** ***

Ctrl_A1H    CGCTTCACCTACATCCCGGGGGCGGCTCGCGCAGCTGCGTGGGCAAGGAGCTGGCGCGAC  180
Cr_A1H_1    ----------------------------GCGCAGCTGCGTGGGCAAGGAGCTGGCGCGAC  151
Cr_A1H_2    CGCTTCACCTCG--------------------CGCTGCGTGGGCAAGGAGCTGGCGCGAC  160
Cr_A1H_3    CGCGTCTC--------------------CTCCCGCTGCGTGGGCAAGGAGCTGGCGCGAC  160
Cr_A1H_4    ----------------------------GCGCAGCTGCGTGGGCAAGGAGCTGGCGCGAC  151
                                             ***************************

Ctrl_A1H    TCATCCTGAGGATCGTGGTGGTGGAGCTGTGCCGGCGCTGCGAGTGGGAGCTGCCCAACG  240
Cr_A1H_1    TCATCCTGAGGATCGTGGTGGTGGAGCTGTGCCGGCGCTGCGAGTGGGAGCTGCCCAACG  211
Cr_A1H_2    TCATCCTGAGGATCGTGGTGGTGGAGCTGTGCCGGCGCTGCGAGTGGGAGCTGCCCAACG  220
Cr_A1H_3    TCATCCTGAGGATCGTGGTGGTGGAGCTGTGCCGGCGCTGCGAGTGGGAGCTGCCCAACG  220
Cr_A1H_4    TCATCCTGAGGATCGTGGTGGTGGAGCTGTGCCGGCGCTGCGAGTGGGAGCTGCCCAACG  211
            ************************************************************
```

```
Ctrl_A1I    CCGCTCACCACTCCCGCCCCCTCCAGGACCTGAAGGAGTCGGCCACGGAGCTCCTGTTCG  420
Cr_A1I      CCGCTCACCACTCCCGCCCCCTCCAGGACCTGAAGGAGTCGGCCACGGAGCTCCTGT--G  419
            *********************************************************  *

Ctrl_A1I    GGGGACACGAGACGACGGCCAGCGCCGCCACGTCACTCGTCATGCACCTGGCAATCCACC  480
Cr_A1I      GGGGACACGAGACGACGGCCAGCGCCGCCACGTCGCTCGTCATGCACCTGGCAATCCACC  478
            ********************************** *************************
```

	Cyp26A1_H	Cyp26A1_I	Cyp26B1/C1a_H
Mutant CRISPR loci	4/7	1/12	5/6
Control CRISPR loci	5/5	6/6	3/3

Supplementary Figure 5: **Genotyping strategy and sequencing results for *Cyp26* CRISPR embryos**
Cartoon illustrating the design of primers for amplifying each site targeted by the *gRNAs* in the *Cyp26* loci. The primer binding sites are indicated in bold purple. For each site, we show an alignment of mutant loci (Cr_) sequences with the sequence of a control locus (Ctrl), the site targeted by the gRNA is indicated in bold and the PAM in red. A table indicating the proportion of mutant CRISPR or control CRISPR loci for each site is shown.

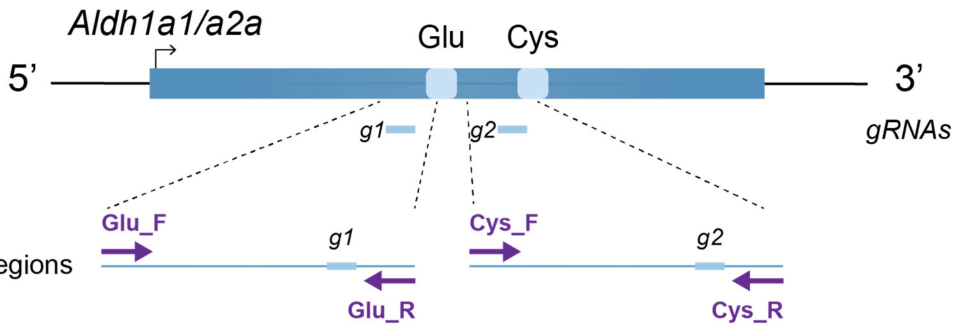

1) PCR amplification of the targeted regions

2) Sequencing of the targeted regions

```
Ctrl_Glu     CCCTTACGTAACGCAGGTGGGGAAGTTGATCCAGGAGGAGGCCGGCAAGAGTAACCTGAA   179
Cr_Glu_1     CCTTTACGTAACGCAGGTGGGGAAGTTGATCCAGGA--------------GTAACGTGAA   73
Cr_Glu_2     CCCTTACGTAACGCAGGTGGGGAAGTTGATCCAGGA--------------GTAACCTGAA   165
Cr_Glu_3     CCCTTACGTAACGCAGGTGGGGAAGTTGATCCAGGA--------------GTAACCTGAA   166
Cr_Glu_4     CCCTTACGTAACGCAGGTGGGGAAGTTGATCCAGGAGTAACCTGAAGC------------   165
Cr_Glu_5     CCCTTACGTAACGCAGGTGGGGAAGTTGATCCAGGA--------------GTAACCTGAA   165
             ** *********************************

Ctrl_Glu     GCGCGTGACGCTGGAGCTTGGCGGGAAGAGCCCCATCATCGTCTTCGCCGACGCCGACCG   239
Cr_Glu_1     GCGAGTGACGCTGAAGCTTGGCGGGAAGAGCCCCATCATCGTATTCGCCGAA-CCGACCG   132
Cr_Glu_2     GCGCGTGACGCTGGAGCTTGGCGGGAAGAGCCCCATCATCGTCTTCGCCGACGCCGACCG   225
Cr_Glu_3     GCGCGTGACGCTGGAGCTTGGCGGGAAGAGCCCCATCATCGTCTTCGCCGACGCCGACCG   226
Cr_Glu_4     ---GCGGACGCTGGAGCGTGGCGGGAAGAGCCCCATCATCGTTTTGACCGACGCCGACCG   222
Cr_Glu_5     GCGCGTGACGCTGGAGCTTGGCGGGAAGAGCCCCATCATCGTCTTCGCCGACGCCGACCG   225
                   ******* *** *********************** **  ****  *******
```

```
Ctrl_Cys     CCCCTGCTGTGACACTGCTGACTCGGCGTGGTTCCCACCCCTCCACCCCCCCCCTCCCAC   240
Cr_Cys_1     CCCCTGCTGTGACACTGCTGACTCGGCGTGGTTCCCACCCCTCCACCCCCCCCCTCCCAC   240
Cr_Cys_2     CCCCTGCTGTGACACTGCTGACTCGGCGTGGTTCCCACCCCTCCACCCCCCCCCTCCCAC   240
Cr_Cys_3     CCCCTGCTGTGACACTGCTGACTCGGCGTGGTTCCCACCCCTCCACCCCCCCC-TCCCAC   240
Cr_Cys_4     CCCCTGCTGTGACACTGCTGACTCGGCGTGGTTCCCACCCCTCCACCCCCCCCCTCCCAC   238
Cr_Cys_5     CCCCTGCTGTGACACTGCTGACTCGGCGTGGTTCCCACCCCTCCACCC------------   238
Cr_Cys_6     CCCCTGCTGTGACACTGCTGACTCGGCGTGGTTCCCACCCCTCCACCC------------   238
Cr_Cys_7     CCCCTGCTGTGACACTGCTGACTCGGCGTGGTTCCCACCCCTCCACCC------------   240
             ************************************************

Ctrl_Cys     TACGAGCAGTGGACGCGGCGGTGGAGCAGGCGCACCAGGGGGTGTTCTGGAACCAGGGCC   300
Cr_Cys_1     -------------------------------------------------------GGGCC   258
Cr_Cys_2     -------------------------------------------------------GGGCC   258
Cr_Cys_3     -------------------------------------------------------GGGCC   249
Cr_Cys_4     -------------------------------------------------------GGGCC   260
Cr_Cys_5     ------------------------------------------------------AGGGCC   250
Cr_Cys_6     ------------------------------------------------------AGGGCC   250
Cr_Cys_7     ------------------------------------------------------AGGGCC   244
                                                                    *****
```

	Aldh1a1/a2a_Glu	Aldh1a1/a2a_Cys
Mutant CRISPR loci	5/5	7/11
Control CRISPR loci	5/5	3/3

Supplementary Figure 6: **Genotyping strategy and sequencing results for *Aldh1a1/a2a* CRISPR embryos.** Cartoon illustrating the design of primers for amplifying each site targeted by the *gRNAs* in the *Aldh1a1/a2a* gene loci. Primer binding sites are indicated in bold purple. For each site, we show an alignment of mutant loci (Cr_) sequences with the sequence of a control locus (Ctrl), the site targeted by the gRNA is indicated in bold and the PAM in red. A table indicating the proportion of mutant CRISPR or control CRISPR loci for each site is shown.

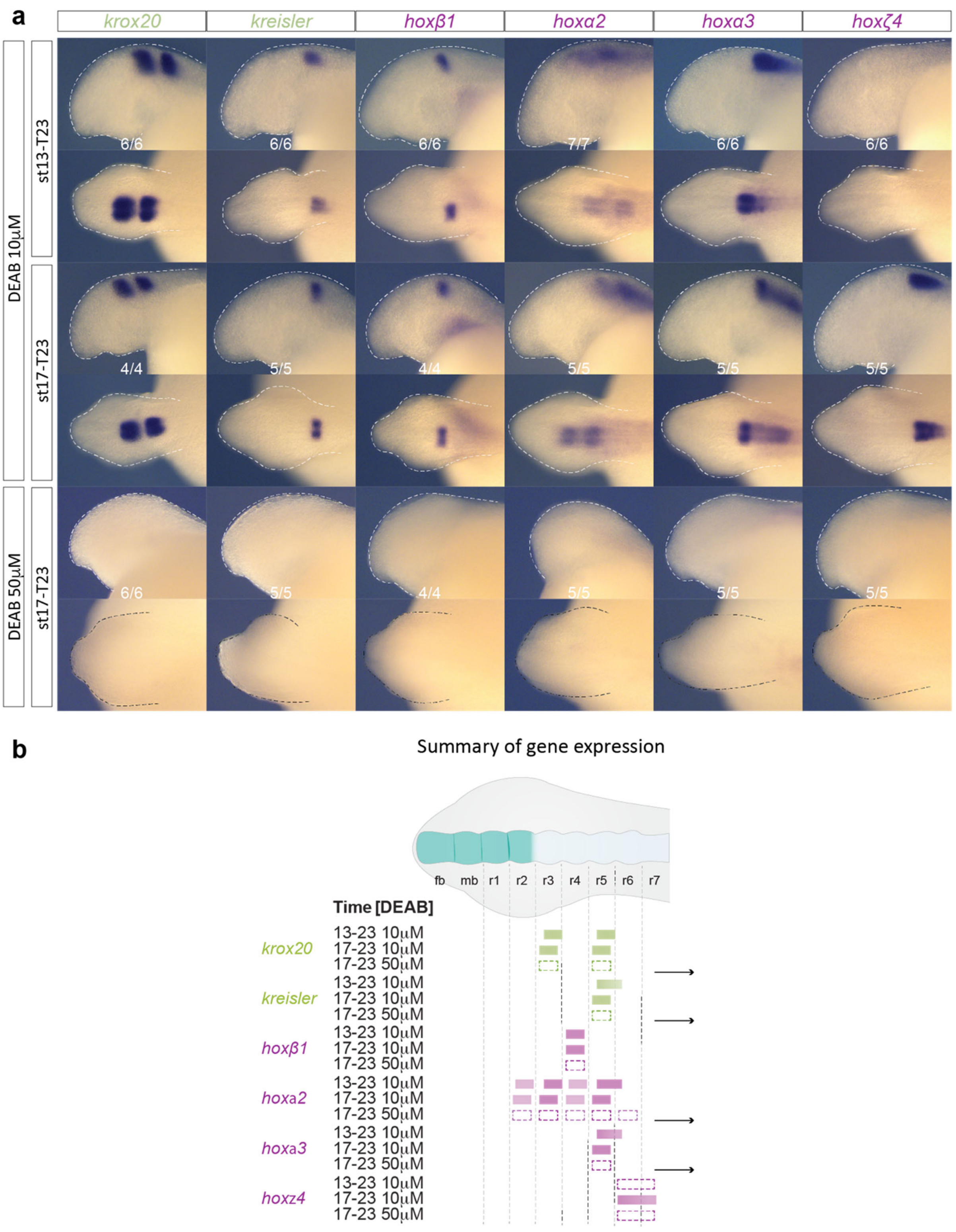

Supplementary Figure 7: **Effect of DEAB concentration and timing of treatment on hindbrain patterning. (a)** cISH of key patterning hindbrain markers in embryos treated with 10 μM of DEAB at gastrulation (st13), 10 μM of DEAB at early neurulation (st17) or 50 μM of DEAB at early neurulation (st17). For each gene, the most representative phenotype is shown, and numbers of experimental replicates are indicated. These molecular phenotypes obtained for embryos treated with 10 μM of DEAB at gastrulation (st13) are less severe than the ones obtained when treating the embryos with 50 μM DEAB (**Fig. 5d**) and are reminiscent of the phenotypes obtained in the Cr *Aldh1a1/a2a* mutants (**Fig. 8c**). **(b)** Cartoon summarizing the effects of different concentrations and timing of treatments on hindbrain patterning.

Mouse (*Mus musculus*): Aldh1a1: NP_001348432.1; Aldh1a2: NP_033048.2; Aldh8a1: NP_848828.1; Cyp26A1: XP_017173542.1; Cyp26B1: NP_001171184.1; Cyp26C1: XP_017173748.1; Cyp51: NP_064394.2

Spotted gar (*Lepisosteus oculatus*): Aldh1a1: XP_015222024.1; Aldh1a2: XP_006628784.1; Aldh8a1: XP_006625886.1; Cyp26A1-LG5: XP_015202021.1; Cyp26B1-LG5: XP_015202022.1; Cyp26B1-LG4: XP_006629157.1; Cyp51: XP_006636075.1

West indian coelacanth (*Latimeria chalumnae*): Aldh1a1: XP_006001725.2; Aldh1a2: XP_005998797.2; Aldh8a1: XP_014352052.1; Cyp26A1: XP_005991616.1; Cyp26B1: XP_005997719.1; Cyp26C1: XP_014341348.1; Cyp51: XP_014350652.1

Whale shark (*Rhincodon typus*): Aldh1a1-like: XP_015222024.1; Aldh1a2: XP_020369553.1; Aldh8a1: XP_048452951.1; Cyp26A1: XP_020382659.1; Cyp26B1: XP_020378695.1; Cyp26C1: XP_020367038.1; Cyp51: XP_020371022.1

Inshore hagfish (*Eptatretus burgeri*): Aldh1a1: ENSEBUG00000010361; Aldh8a1: ENSEBUG00000011145; Cyp26C1 (10538): ENSEBUG00000010538; Cyp26C1 (06909): ENSEBUG00000006909; Cyp51: ENSEBUG00000006843

Sea lamprey (*Petromyzon marinus*): Aldh1a1/a2a (Chr1): XP_032805864.1; Aldh1a1/a2b (Ch40): XP_032824682.1; Aldh8a1: XP_032810842.1; Cyp26A1: XP_032808019.1-2; Cyp26B1/C1a (Chr11): XP_032808017.1; Cyp26B1/C1b (Chr9): XP_032806163.1; Cyp51: XP_032816590.1

Thorny Skate (*Amblyraja radiata*): Aldh1a1-like: XP_032873441.1; Aldh1a2: XP_032906516.1; Cyp26A1: XP_032889480.1; Cyp26B1: XP_032885953.1; Cyp26C1: XP_032889482.1;

Chicken (*Gallus gallus*): Aldh1a1: NP_989908.2; Aldh1a2: NP_001384737.1; Cyp26A1: NP_001001129.2; Cyp26B1: XP_015141554.1; Cyp26C1: XP_421678.6;

Chinese soft-shelled turtle (*Pelodiscus sinensis*): Aldh1a1: XP_006137885.1; Aldh1a2: XP_006112531.1; Cyp26A1: XP_006113245.1; Cyp26B1: XP_025042348.1; Cyp26C1: XP_025035575.1;

West African lungfish (*Protopterus annectens*): Aldh1a1: XP_043916765.1; Aldh1a2: XP_043934839.1; Cyp26A1: XP_043912621.1; Cyp26B1: XP_043919907.1; Cyp26C1: XP_043912622.1;

Reedfish (*Erpetoichthys calabaricus*): Aldh1a1: XP_028658993.1; Aldh1a2: XP_028678887.1; Cyp26A1: XP_028651199.1; Cyp26B1: XP_028657295.1; Cyp26C1: XP_028651196.1

Amphioxus (*Branchiostoma floridae*): BfAldh1a1/a2a: XP_035683184.1; BfAldh1a1/a2b: XP_035666435.1; BfAldh1a1/a2c: XP_035683791.1; BfAldh1a1/a2d: XP_035683789.1; BfAldh1a1/a2e: XP_035659507.1; BfAldh1a1/a2f: XP_035659462.1; BfCyp26-1: LOC118413434; BfCyp26-2: XP_035672703.1; BfCyp26-3: XP_035672700.1;

Supplementary Figure 8: Protein accession numbers used for phylogeny analysis. Protein sequences corresponding to each gene were retrieved using both ENSEMBL and NCBI databases, focusing on the latest available version of the genome and/or the most complete available genome annotation. Amphioxus *Cyp26* and *Aldh1* gene families were used as outgroups for the analyses of *Cyp26* and *Aldh1a* complements.

Mouse (*Mus musculus*): *Aldh1a1*: NM_001361503.1; *Aldh1a2*: NM_009022.4;
Cyp26A1: XM_017318053.2; *Cyp26B1*: NM_001177713.1; *Cyp26C1*: XM_017318259.3

Grey short-tailed opossum (*Monodelphis domestica*): *Aldh1a1*: XM_001373091.4;
Aldh1a2: XM_007479682.2; *Cyp26A1*: XM_001375255.4; *Cyp26C1*: XM_007478798.1

Platypus (*Ornithorhynchus anatinus*): *Aldh1a1*: XM_007667019.3;
Aldh1a2: XM_029070050.1; *Cyp26B1*: XM_029046562.2; *Cyp26C1*: XM_029060242.1

Chicken (*Gallus gallus*): *Aldh1a1*: NM_204577.5; *Aldh1a2*: NM_001397808.1;
Cyp26A1: XM_015288598.4; *Cyp26B1*: XM_015286068.4; *Cyp26C1*: XM_421678.8

Spotted gar (*Lepisosteus oculatus*): *Aldh1a1*: XM_015366538.1; *Aldh1a2*: XM_006628721.2;
Cyp26A1LG5: XM_015346535.1; *Cyp26B1-LG5*: XM_015346536.1; *Cyp26B1-LG4*: XM_006629094.2

West indian coelacanth (*Latimeria chalumnae*): *Aldh1a1*: XM_006001663.2;
Aldh1a2: XM_005998735.2; *Cyp26A1*: XM_005991554.1; *Cyp26B1*: XM_005997657.2;
Cyp26C1: XM_014485862.1

Whale shark (*Rhincodon typus*): *Aldh1a1-like*: XM_020527364.2; *Aldh1a2*: XM_020513964.2;
Cyp26A1: XM_048608684.1; *Cyp26B1*: XM_020523106.1; *Cyp26C1*: XM_020511449.1

Sea lamprey (*Petromyzon marinus*): *Aldh1a1/a2a* (Chr1): XM_032949973.1;
Aldh1a1/a2b (Chr40): XM_032968791.1; *Cyp26A1*: XM_032952128.1;
Cyp26B1/C1a (Chr11): XM_032952126.1; *Cyp26B1/C1b* (Chr9): XM_032950272.1

Supplementary Figure 9: mRNA accession numbers used for synteny analysis. mRNA accession numbers of *Cyp26* and *Aldh1a* used to conduct the synteny analyses in different vertebrate models.

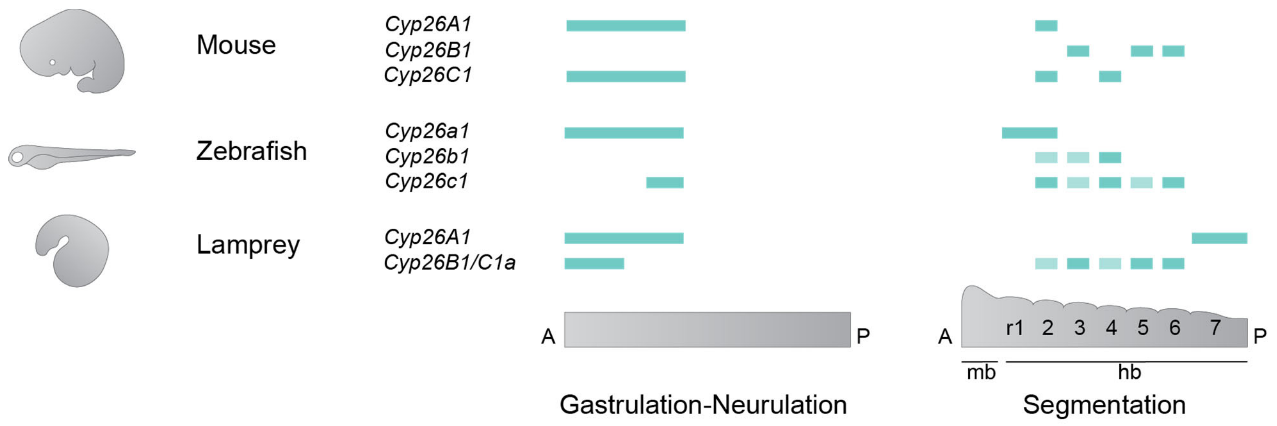

Supplementary Figure 10: comparison of *Cyp26* expression developmental dynamics in different vertebrates. Schematic representation of the dynamic changes in *Cyp26* expression during early processes of development (gastrulation-neurulation) and during hindbrain segmentation in mouse, zebrafish and lamprey, illustrating both similarities and differences between orthologues.

Species	Exons	AA
Pm Cyp26A1	7 exons	497 AA
Pm Cyp26B1/C1a	6 exons	529 AA
Pm Cyp26B1/C1b	6 exons	634 AA
Eb Cyp26 06909	6 exons	468 AA
Eb Cyp26 10538	6 exons	528 AA

Supplementary Table 1: Cyp26 gene structure and protein length in cyclostomes. Pm, *Petromyzon marinus* (sea lamprey); Eb, *Eptatretus burgeri* (inshore hagfish); AA. Amino-acid.

Species	*Cyp26A1*	Cyp26A1	*Cyp26B1*	Cyp26B1	*Cyp26C1*	Cyp26C1
Mm	7 exons	497 AA	6 exons	512 AA	6 exons	518 AA
Oa	NF		6 exons	511 AA	6 exons	571 AA
Lo	7 exons	492 AA	6 exons	511 AA	6 exons	554 AA
Lc	7 exons	493 AA	6 exons	511 AA	6 exons	634 AA
Rt	7 exons	494 AA	6 exons	511 AA	NF	
Cc					6exons	560 AA

Supplementary Table 2: Cyp26 gene structure and protein length in jawed vertebrates. Mm, *Mus musculus* (mouse); *Oa, Ornithorhynchus anatinus* (platypus)*; Lo, Lepisosteus oculatus* (spotted gar)*;* Lc, *Latimeria chalumnae* (coelacanth); Rt, *Rhincodon typus* (whale shark); *Cc, Carcharodon carcharias* (great white shark); NF: not found; AA. Amino-acid.

Species	Exons	AA
Pm Aldh1a1/a2a	13	520 AA
Pm Aldh1a1/a2b	13	512AA or 459 AA
Eb Aldh1a1	13	515 AA

Supplementary Table 3: Aldh1a gene structure and protein length in cyclostomes. Pm, *Petromyzon marinus* (sea lamprey); Eb, *Eptatretus burgeri* (inshore hagfish); AA. Amino-acid.

Species	*Aldh1a1*	Aldh1a1	*Aldh1a2*	Aldh1a2
Mm	13 exons	501 AA	13 exons	518 AA
Oa	13 exons	511 AA	13 exons	518 AA or 422 AA
Lo	13 exons	519 AA or 527 AA	13 exons	518 AA
Lc	13 exons	514	13 exons	518 AA or 422 AA
Rt	13 exons	510 or 537 AA	13 exons	518 AA

Supplementary Table 4: Aldh1a gene structure and protein length in jawed vertebrates. Mm, *Mus musculus* (mouse); *Oa, Ornithorhynchus anatinus* (platypus); *Lo, Lepisosteus oculatus* (spotted gar); Lc, *Latimeria chalumnae* (coelacanth); Rt, *Rhincodon typus* (whale shark); AA. Amino-acid.

Gene	Accession Number	Probe set size	Amplifier/AlexaFluor	Average Probe Concentration
Aldh1a1/a2a	LOC116940317	19	B3-488	6 nM
Cyp26A1	LOC116941264	23	B4-546	5 nM
Cyp26B1/C1a	LOC116941263	22	B1-647	5 nM
krox20 (egr1)	LOC116940673	18	B3-488	6 nM
kreisler (mafB-like)	LOC116954108	12	B4-546	11 nM
hoxβ1	LOC103091820	13	B1-647	8 nM

Supplementary Table 5: HCR FISH probe set design. Summary of the probe set design obtained for each gene targeted by FISH HCR; nM: nanomolar.

name	5'--> 3' sequence	description	product size
A1_H_F	TACAGCATCAAGGAGACGCA	Cyp26A1_H helix	405 bp
A1_H_R	CGTCTTGTGCTGGCATAAATG		
A1_I_F	GCTGAGACAAAGCGGGAAAG	Cyp26A1_I helix	568 bp
A1_I_R	CCAGGCGAGGGAGATAACAT		
**name**	**5'--> 3' sequence**	**description**	**product size**
C1_H_F	GGAGCGTCCTCTACAGCATC	Cyp26C1_H helix	480 bp
C1_H_R	CTCCTCCGTCTCTCTGTCGT		
**name**	**5'--> 3' sequence**	**description**	**product size**
Glu_F	TGGCGGTCGATTCTTCTCTT	Glutamate site genotyping	360 bp
Glu_R	TATTGGCTGCGCAATCTCAC		
Cys_F	ATATACAGCAGGCGTCAGGG	Cysteine site genotyping	355 bp
Cys_R	CGCACGAACTCGTCGTAGA		

Supplementary Table 6: Primers for CRISPR genotyping. Primers used for genotyping targeted regions in CRISPR embryo; F: Forward, R: Reverse.
