# Supplementary material for: Sea lamprey enlightens the origin of the coupling of retinoic acid signaling to vertebrate hindbrain segmentation
Source: Nat Commun. 2024 Feb 20;15:1538. doi: 10.1038/s41467-024-45911-x (PMC10879103; doi:10.1038/s41467-024-45911-x)
Supplement: Supplementary file 3 — Reporting Summary [file 41467_2024_45911_MOESM3_ESM.pdf]

nature portfolio

Corresponding author(s): Robb Krumlauf NCOMMS-23-30143A

Last updated by author(s): Jan 29, 2024

Reporting Summary

Nature Portfolio wishes to improve the reproducibility of the work that we publish. This form provides structure for consistency and transparency in reporting. For further information on Nature Portfolio policies, see our Editorial Policies and the Editorial Policy Checklist.

Statistics

For all statistical analyses, confirm that the following items are present in the figure legend, table legend, main text, or Methods section.

n/a	Confirmed	
☐	☒	The exact sample size (*n*) for each experimental group/condition, given as a discrete number and unit of measurement
☒	☐	A statement on whether measurements were taken from distinct samples or whether the same sample was measured repeatedly
☒	☐	The statistical test(s) used AND whether they are one- or two-sided<br>*Only common tests should be described solely by name; describe more complex techniques in the Methods section.*
☒	☐	A description of all covariates tested
☒	☐	A description of any assumptions or corrections, such as tests of normality and adjustment for multiple comparisons
☒	☐	A full description of the statistical parameters including central tendency (e.g. means) or other basic estimates (e.g. regression coefficient) AND variation (e.g. standard deviation) or associated estimates of uncertainty (e.g. confidence intervals)
☒	☐	For null hypothesis testing, the test statistic (e.g. *F*, *t*, *r*) with confidence intervals, effect sizes, degrees of freedom and *P* value noted<br>*Give P values as exact values whenever suitable.*
☒	☐	For Bayesian analysis, information on the choice of priors and Markov chain Monte Carlo settings
☒	☐	For hierarchical and complex designs, identification of the appropriate level for tests and full reporting of outcomes
☒	☐	Estimates of effect sizes (e.g. Cohen's *d*, Pearson's *r*), indicating how they were calculated

Our web collection on statistics for biologists contains articles on many of the points above.

Software and code

Policy information about availability of computer code

Data collection	Sea lamprey (Petromyzon marinus) Aldh1a and Cyp26 gene sequences were retrieved from the JBrowse browser and NCBI database, using the most-recent version of the Sea lamprey germline genome (KPetMar1). Cyp26 and Aldha1 protein sequences corresponding to each gene were retrieved using both ENSEMBL and NCBI, focusing on the latest available version of the genome and/or the most complete available genome annotation for each species. RNAseq profiles corresponding to 1 to 5-day post fertilization (dpf) as well as stages st18 to st24 (Tahara stages) obtained from P. marinus dorsal neural tube tissues and/or whole embryos and accessible on the KPetMar1 genome browser. Colorimetric images were taken on a Leica MZ APO microscope using the Lumenera Infinity 3 camera at a magnification of 50X together with the Infinity Analyze software. HCR images were taken on a Nikon AT-AT inverted confocal microscope, using a 4X magnification for lateral imaging of the developing head (st20 embryos) and a 10X magnification for dorsal imaging of the developing head (st21-st23.5 embryos). Alignments for design of CRISPR experiments were made using Clustal Omega Multiple Sequence Alignment tool. Conserved amino acids were highlighted in yellow on Snapgene, using a threshold of 95% for conservation.
Data analysis	Protein sequences were aligned using the CLUSTALW protein alignment algorithm in MEGA11 and their evolutionary history was inferred using the Maximum Likelihood Tree method and WAG model. We investigated the chromosomal organization of genes (i.e., synteny) located in the vicinity of Cyp26 and Aldh1a genes in different vertebrate models using both ENSEMBL and NCBI Genome Data Viewer. RNA In Situ probes for P. marinus Aldh1a and Cyp26 genes were designed based on gene sequences predicted by the Refseq model of KPetmar1. HCR images were analyzed and post processed for brightness and contrast in Fiji-ImageJ and Adobe Photoshop 2023, then cropped and assembled into montages in Adobe Illustrator 2023. For CRISPR experiments all gRNAs were designed using CRISPOR with KPetMar1 as a target reference genome and the standard -NGG PAM motif.

For manuscripts utilizing custom algorithms or software that are central to the research but not yet described in published literature, software must be made available to editors and reviewers. We strongly encourage code deposition in a community repository (e.g. GitHub). See the Nature Portfolio guidelines for submitting code & software for further information.

Data

Policy information about availability of data

All manuscripts must include a data availability statement. This statement should provide the following information, where applicable:

- Accession codes, unique identifiers, or web links for publicly available datasets
- A description of any restrictions on data availability
- For clinical datasets or third party data, please ensure that the statement adheres to our policy

All data supporting the findings of this study are available within the article and its supplementary information files or from the corresponding author upon reasonable request. Original data and images underlying this manuscript can be accessed from the Stowers Original Data Repository at http://www.stowers.org/research/publications/libpb-2405

Research involving human participants, their data, or biological material

Policy information about studies with human participants or human data. See also policy information about sex, gender (identity/presentation), and sexual orientation and race, ethnicity and racism.

Reporting on sex and gender	N/A
Reporting on race, ethnicity, or other socially relevant groupings	N/A
Population characteristics	N/A
Recruitment	N/A
Ethics oversight	N/A

Note that full information on the approval of the study protocol must also be provided in the manuscript.

Field-specific reporting

Please select the one below that is the best fit for your research. If you are not sure, read the appropriate sections before making your selection.

☒ Life sciences ☐ Behavioural & social sciences ☐ Ecological, evolutionary & environmental sciences

For a reference copy of the document with all sections, see nature.com/documents/nr-reporting-summary-flat.pdf

Life sciences study design

All studies must disclose on these points even when the disclosure is negative.

Sample size	Sample sizes for the various types of experimental approaches were not calculated by a statistical method. For gene expression by in situ hybridization, drug treatment and CRISPR experiments we desired at least 5-10 embryos for each stage, numbers of probes sets, treatment conditions or individual assays to be analyzed. In addition, because some phenotypes might be complex and require additional analysis we also want to have a few extra groups of 5-10 embryos in reserve for other analyses. These parameters served to estimate the number of embryos required for the experiments. This number of embryos were cultured and or treated in large batches at the same time to ensure continuity of results between experimental approaches and assays. In CRISPR and transgenic regulatory analyses in lamprey embryos, in general a minimum of 100 embryos were injected for each construct. This permits monitoring efficiency and variability for each construct due to mosaicism and position effects of integration.
Data exclusions	No data was excluded from the analyses.
Replication	To monitor reproducibility in regulatory and CRISPR analyses a minimum of 100 embryos were injected and results for all embryos scored. All replicates of these experiments yielded similar results.
Randomization	Experimental groups were assigned based on CRISPR/Cas9 gRNA and DNA constructs used for injection and for the specific drug treatments and control embryos.
Blinding	Investigators were not blinded to groups during data collection and allocation. The analysis of gene expression patterns, morphological and molecular phenotypes and other properties of collected embryos from various experimental conditions were independently scored by two people. Given that there is mosaicism in the embryos, it is essential to have a trained eye evaluate the embryos generated on different days in order to generate interpretable results.

Reporting for specific materials, systems and methods

We require information from authors about some types of materials, experimental systems and methods used in many studies. Here, indicate whether each material, system or method listed is relevant to your study. If you are not sure if a list item applies to your research, read the appropriate section before selecting a response.

Materials & experimental systems

n/a	Involved in the study
☒	☐ Antibodies
☒	☐ Eukaryotic cell lines
☒	☐ Palaeontology and archaeology
☐	☒ Animals and other organisms
☒	☐ Clinical data
☒	☐ Dual use research of concern
☒	☐ Plants

Methods

n/a	Involved in the study
☒	☐ ChIP-seq
☒	☐ Flow cytometry
☒	☐ MRI-based neuroimaging

Animals and other research organisms

Policy information about studies involving animals; ARRIVE guidelines recommended for reporting animal research, and Sex and Gender in Research

Laboratory animals	No standard laboratory animals were used in these experiments.
Wild animals	Adult lamprey are used for breeding purposes and are >7 years of age. Gravid male and female sea lamprey (Petromyzon marinus) were caught in the wild and provided by the Great Lakes Fisheries Commission (GLFC), in coordination with the USGS Hammond Bay Biological Station, Millersburg, MI, USA. They were sent overnight, in accordance with CA state import permits, in chilled, oxygenated water to the lamprey facility at the California Institute of Technology. They were maintained under the parameters set in accordance with the Guide for the Care and Use of Laboratory Animals of the National Institutes of Health, with protocols approved by the Institutional Animal Care and Use Committees of the California Institute of Technology (lamprey, Protocol #1436-17). After spawning, the captive adult lamprey died of natural causes, as per their semelparous reproductive strategy.
Reporting on sex	We use an equal number of males and females for producing embryos. We are not able to determine the gender of embryos since the gonads do not develop until much later than the stages under consideration. Hence, information on the sex of embryos was not collected or considered in the study. Samples of developing embryos were analyzed at relatively early stages and there is no convenient or robust method for determining the sex of embryos during these developmental stages. We have no reason to believe that this will bias or impact interpretation of the analyses.
Field-collected samples	Sea lamprey embryos were harvested by in-vitro fertilization at the California Institute of Technology lamprey facility, using captive gravid lamprey (Petromyzon marinus) obtained from the wild and provided by the Great Lakes Fisheries Commission (GLFC), in coordination with the USGS Hammond Bay Biological Station, Millersburg, MI, USA. Captive gravid lamprey were maintained at an appropriate temperature (10-18 degrees C) and on a 15:9 (Day:Night) photoperiod. Adult lamprey naturally die at the end of their reproductive period. Any animals that are sick are euthanized by protocols approved by the Institutional Animal Care and Use Committee (IACUC). After spawning, the captive adult lamprey died of natural causes, as per their semelparous reproductive strategy. The in vitro fertilized embryos were cultured in 0.05X Marc's Modified Ringers solution (MMR) embryo media (18 °C) plus or minus drugs, harvested at the desired Tahara stages and then sacrificed by fixation in 1X MEMFA (1-part 10X MEMFA Salts, 1-part 37% formaldehyde, 8-parts H_2O).
Ethics oversight	Gravid male and female sea lamprey (Petromyzon marinus) were caught in the wild and provided by the Great Lakes Fisheries Commission (GLFC), in coordination with the USGS Hammond Bay Biological Station, Millersburg, MI, USA. They were sent overnight, in accordance with CA state import permits, in chilled, oxygenated water to the lamprey facility at the California Institute of Technology. They were maintained under the parameters set in accordance with the Guide for the Care and Use of Laboratory Animals of the National Institutes of Health, with protocols approved by the Institutional Animal Care and Use Committees of the California Institute of Technology (lamprey, Protocol IA23-1436).

Note that full information on the approval of the study protocol must also be provided in the manuscript.
